# Diverse Lineages of *Candida albicans* Live on Old Oaks

**DOI:** 10.1534/genetics.118.301482

**Published:** 2018-11-21

**Authors:** Douda Bensasson, Jo Dicks, John M. Ludwig, Christopher J. Bond, Adam Elliston, Ian N. Roberts, Stephen A. James

**Affiliations:** * Department of Plant Biology, University of Georgia, Athens, Georgia 30602; † Institute of Bioinformatics, University of Georgia, Athens, Georgia 30602; ‡ National Collection of Yeast Cultures, Quadram Institute Bioscience, Norwich NR4 7UA, UK

**Keywords:** Population genomics, loss of heterozygosity (LOH), molecular evolution, yeast ecology, environmental reservoir, clonality, asexual

## Abstract

The human pathogen *Candida albicans* is considered an obligate commensal of animals, yet it is occasionally isolated from trees, shrubs, and grass. We generated genome sequence data for three strains of *C. albicans* that we isolated from oak trees in an ancient wood pasture, and compared these to the genomes of over 200 clinical strains. *C. albicans* strains from oak are similar to clinical *C. albicans* in that they are predominantly diploid and can become homozygous at the mating locus through whole-chromosome loss of heterozygosity. Oak strains differed from clinical strains in showing slightly higher levels of heterozygosity genome-wide. Using phylogenomic analyses and *in silico* chromosome painting, we show that each oak strain is more closely related to strains from humans and other animals than to strains from other oaks. The high genetic diversity of *C. albicans* from old oaks shows that they can live in this environment for extended periods of time.

## Abstract (precis)

Most humans are inhabited by the yeast *Candida albicans* at some point. While largely harmless, it is the most common cause of yeast infections. Though previously unclear whether the yeast can live outside of warm-blooded animals, Bensasson *et al.*....

THE yeast *Candida albicans* is the most common yeast pathogen in humans (Barnett 2008), yet it is also a commensal in most humans and inhabits a broad range of warm-blooded animals (Barnett 2008). Unlike other *Candida* species, *C. albicans* is only rarely isolated from plants or other environmental substrates (Barnett 2008; Lachance *et al.* 2011) and is generally considered an “obligate commensal” (Hall and Noverr 2017). There were early discoveries of *C. albicans* on gorse flowers and myrtle leaves on a hillside grazed by sheep and goats in Portugal (van Uden *et al.* 1956), and on grass in a pasture in New Zealand (Di Menna 1958). More recently it was isolated from a beetle and an African tulip tree in the Cook Islands (Lachance *et al.* 2011), and we isolated *C. albicans* from oak trees in an ancient wood pasture in the United Kingdom (Robinson *et al.* 2016).

Many species of yeast live on trees, including other opportunistic pathogenic *Candida* species such as *Candida tropicalis* and *Candida parapsilosis* (Maganti *et al.* 2011; Carvalho *et al.* 2014). Genetic data shows that *Saccharomyces cerevisiae*, which can also infect humans, stably persists in wild woodland habitats (Fay and Benavides 2005; Aa *et al.* 2006), and the high genomic diversity of *S. cerevisiae* from Asia shows that forests are its ancestral habitat (Peter *et al.* 2018). Indeed, woodlands probably represent the ancestral habitat for all *Saccharomyces* species (Eberlein *et al.* 2015). Forests may also be an ancestral habitat and reservoir for other human fungal pathogens such as *Cryptococcus neoformans* and *Cryptococcus gattii* (May *et al.* 2016; Gerstein and Nielsen 2017).

The isolation of *C. albicans* from plants (van Uden *et al.* 1956; Di Menna 1958; Lachance *et al.* 2011; Robinson *et al.* 2016) raises the question of whether *C. albicans* is truly an obligate commensal of warm-blooded animals. Laboratory experiments show that *C. albicans* can grow and mate at room temperature (Hull *et al.* 2000; Magee and Magee 2000;), and it retains an intact aquaporin gene whose only known phenotype is freeze tolerance (Tanghe *et al.* 2005). It is therefore possible that *C. albicans* populations could survive away from warm-blooded animals.

Here, we generated genome sequences for three strains of *C. albicans* from oak bark from the New Forest in the United Kingdom (Robinson *et al.* 2016). These are the first *C. albicans* genomes sequenced from a nonanimal source, and we compared them to the genomes of over 200 strains from humans and animals (Muzzey *et al.* 2013; Hirakawa *et al.* 2015; Ropars *et al.* 2018). Although they were collected from the same woodland, these oak strain genomes are genetically diverged from one another, and are more similar to diverged clinical lineages than they are to each other. However, oak strains differ from clinical strains in showing higher levels of genome-wide heterozygosity, suggesting that they were subject to different selection or mutation pressures. The genetic diversity of *C. albicans* in this woodland implies that they have lived there long enough for distinct lineages to accumulate on unusually old trees. Therefore, *C. albicans* can live in the oak environment for prolonged periods.

## Materials and Methods

### Yeast strains and genome data

We generated short-read genome data for the type strain of *C. albicans* (NCYC 597) and three strains of *C. albicans* from the bark of oak trees in the New Forest in the United Kingdom (Robinson *et al.* 2016; Table 1). Robinson *et al.* (2016) describe the methods used to isolate the oak strains. Negative controls were generated at every field site, and these were subjected to the same procedures and handling as other samples except that no bark was inserted into negative controls after opening tubes in the field. Two of the three trees with *C. albicans* (FRI5 and FRI10) were directly associated with negative controls and there were negative controls at 6 of the 30 trees sampled in the New Forest (FRI5, FRI10, FRI15, OCK5, OCK10, and OCK15). In total, six out of the 125 sample tubes collected in the New Forest (Robinson *et al.* 2016) were negative controls and all of them were clear.

**Table 1:** Three *C*. *albicans* isolates from English and sessile oaks in the new forest in the United Kingdom

Strain*^a^*	Alternate name	Latitude and longitude	Trunk girth (m)*^b^*	Other yeast species from the same tree
NCYC 4144	FRI10b.1	50.92785 −1.657083	4.12	*Lachancea thermotolerans*
NCYC 4145	FR11a.1	50.928483 −1.655183	3.88	*Saccharomyces paradoxus* and *Kazachstania servazzii*
NCYC 4146	FRI5d.SM.1	50.928067 −1.656	2.83	None
	FRI and OCK sites	27 oaks, New Forest*^a^*	0.65–3.79*^c^*	*Saccharomyces paradoxus* (11 isolates), *Lachancea thermotolerans* (4 isolates), *Wickerhamomyces anomalus* (2 isolates), *Saccharomycodes ludwigii* (2 isolates), *Debaryomyces hansenii*, *Hyphopichia burtonii*, *Kazachstania servazzii*, *Hanseniaspora osmophila*

a Information from Robinson *et al.* (2016). NCYC 4144 and NCYC 4145 were isolated from sessile oaks (*Quercus petraea*) and NCYC 4146 was isolated from English oak (*Quercus robur*).

b Assuming average United Kingdom woodland boundary conditions for sessile and English oaks, these trunk girths suggest tree ages of ∼220 years old (FRI10), 200 years old (FRI11), and 130 years old (FRI5) according to the guidelines at http://www.wdvta.org.uk/pdf/Estimating-the-age-of-trees.pdf.

c Twenty-five trees had uncoppiced trunk girth estimates. These were mostly smaller than those with *C. albicans* (Wilcoxon test, P=0.04).

The type strain of *C. albicans* (NCYC 597) and the three oak strains were characterized biochemically, morphologically, and physiologically according to the standard methods described by Kurtzman *et al.* (2011) (Supplemental Material, Table S1).

We used the reference genome sequence for *C. albicans* strain SC5314_A22 (haplotype A, version 22; GCF_000182965.3) from the NCBI reference sequence database. For comparisons of oak strain to clinical strain genomes, we used short-read genome data from the European Nucleotide Archive (ENA) for wild-type SC5314 (SRR850113) and the related 1AA mutant (strain AF9318-1, SRR850115; Legrand *et al.* 2008; Muzzey *et al.* 2013), 20 clinical strains (PRJNA193498; Hirakawa *et al.* 2015) (Table S2), and 182 clinical strains (PRJNA432884; Ropars *et al.* 2018) (Table S3).

### Whole-genome sequencing and base calling

Purified genomic DNA was extracted from a 10 ml culture, in a 1.5 ml initial suspension using a MasterPure yeast DNA purification kit (Epicentre) and following the manufacturer’s instructions. Whole-genome sequencing of four *C. albicans* genomic DNA samples (NCYC 4144, NCYC 4145, NCYC 4146, and NCYC 597) was carried out at the Earlham Institute (Norwich, UK). Libraries were constructed using their Low Input Transposase-Enabled (LITE) methodology for library construction of small eukaryotic genomes based on the Illumina Nextera kits. Each library pool was sequenced with a 2 × 250 bp read metric over six lanes of an Illumina HiSeq2500 sequencer. Adapters were trimmed using Trimmomatic (version 0.33; Bolger *et al.* 2014) with default settings for paired-end data and the ILLUMINACLIP tool (2:30:10). We used FastQC (version 0.11.4; http://www.bioinformatics.babraham.ac.uk/projects/fastqc/) to check read quality and the presence of adapters before and after trimming, and used trimmed, paired-read data in subsequent analyses.

Short-read data for all strains [three oak strains, the type strain, 182 strains from Ropars *et al.* (2018), 20 clinical strains from Hirakawa *et al.* (2015), SC5314 and the 1AA mutant from Muzzey *et al.* (2013)] were mapped to the SC5314_A22 reference genome using Burrows–Wheeler Aligner (bwa mem, version 0.7.10; Li and Durbin 2009). We used SAMtools (version 1.2; Li *et al.* 2009) to generate sorted bam files that were merged in cases where there were multiple sets of read-pair data files per strain. To generate a consensus (in genomic variant call format, gVCF) we used mpileup from SAMtools and then bcftools call (with the -c option). SAMtools mpileup was used with default settings except that maximum read depth was increased to 10,000 reads and we used the -I option so insertions and deletions were excluded.

**Figure 1:**
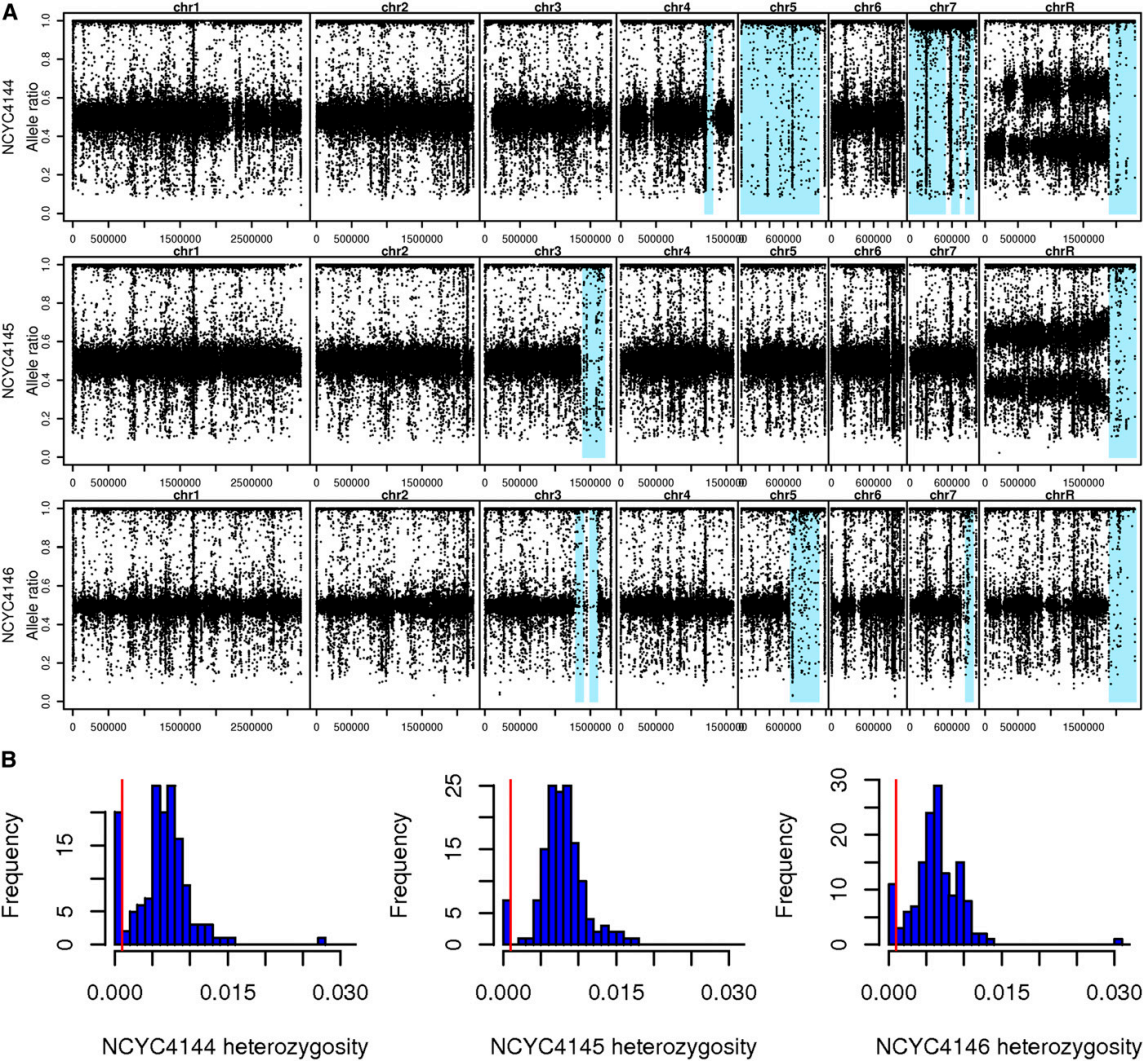
*C. albicans* from oak are mostly diploid with some loss of heterozygosity (LOH). A. The proportion of base calls differing from the reference strain (allele ratios) are mostly 1.0 or 0.5 for oak strains (NCYC 4144-6), suggesting diploidy. Regions that recently homozygozed are shaded light blue. The points that occur in these LOH regions often correspond to the locations of known repeats where short reads are probably mismapped. The oak strain with a/a at its mating locus (NCYC 4144) arrived at this state by LOH for the whole of chromosome 5. B. The proportion of heterozygous sites in 100 kb nonoverlapping windows are either high or low for oak strains (NCYC 4144, NCYC 4145, and NCYC 4146). For oak strains (shown here) and clinical strains (Figure S2) we see two modes; heterozygosity is either low (below the red line at 0.1%), or high (with a mean > 0.4%). Regions with >0.1% heterozygous sites in a 100 kb window were classed as LOH regions and are shown in light blue in A.

To estimate levels of heterozygosity either genome-wide (Table 2) or in 100 kb nonoverlapping windows (Figure 1B), we estimated the proportion of sites that were heterozygous. For all estimates of levels of heterozygosity, only high-quality sites (phred-scaled quality > 40) were considered. Sites were considered heterozygous if the proportion of sites differing from the reference sequence (the allele ratio) was between 0.2 and 0.8. In a diploid, it is also possible for sites to be heterozygous with an allele ratio of 1.0 in cases where three alleles exist for a site because both alleles could differ from that of the reference genome. For example, the reference genome may have an A at a site, and a study strain could show an allele ratio of 1.0 while being heterozygous for C and T alleles. However, levels of intraspecific genetic diversity are sufficiently low that we expect triallelic sites to represent a small proportion of all heterozygous sites, and therefore not to affect our conclusions. If the true proportion of heterozygous sites is 0.007 (close to the levels we observe in Table 2), then the expected proportion of sites with a second point substitution would be $4.9\times 10^{-5}$ (*i.e.*, 0.007^2^). The observed number of high-quality triallelic sites in each (14 Mbp) genome sequence are slightly lower than expected: up to $1\times 10^{-5}$ (144 sites) for the oak strains and all clinical strains except the type strain. The type strain (NCYC 597), which is mostly triploid, has the largest number of triallelic sites (173 sites). Differences between oak and clinical strains in the exclusion of these few sites cannot explain the higher levels of heterozygosity seen for oak strains. exceeding that of clinical strains by thousands of sites.

**Table 2:** *C. albicans* from oak are more heterozygous than most clinical strains from Ropars *et al.* (2018)

Strain or clade	MTL	Mean heterozygosity (maximum–minimum)*^a^*	Filtered length (Mbp)*^b^*	Mean filtered heterozygosity*^c^* (maximum–minimum)
NCYC 4146, clade 4	a/α	0.0062	11.1	0.0066
NCYC 4144, clade18	a/a	0.0061	9.7	0.0068
NCYC 4145, unknown	a/α	0.0077	11.4	0.0078
**3 oak strains***^d^*		**0.0066**	10.7	**0.0070**
**180 clinical strains***^d^* from 17 clades	176a/α,	**0.0048***^e^* (0.0024–0.0071)	9.9	**0.0056***^f^* (0.0032–0.0073)
	3α/α,			
	1a/a			
27 clade 4 strains		0.0053 (0.0045–0.0060)	10.2	0.0062 (0.0060–0064)
4 clade 18 strains		0.0059 (0.0053–0.0065)	10.7	0.0062 (0.0060–0066)
10 unknown strains		0.0057 (0.0048–0.0070)	10.5	0.0063 (0.0054–0071)

Rows in bold show data summarized for all 3 oak strains and 180 clinical strains from Ropars *et al*. (2018).

a Heterozygosity was estimated genome-wide (from ∼14 million high-quality sites) for each strain as the proportion of high-quality sites where 20–80% of reads differed from the reference sequence. Where multiple strains are considered, we show the mean, maximum, and minimum levels of genome-wide heterozygosity.

b The length of genome sequence after excluding loss of heterozygosity (LOH) regions, known repeats, putatively repetitive regions, and centromeres.

c The proportion of heterozygous sites in filtered regions.

d Rows in bold show data summarized for all 3 oak strains and 180 clinical strains from Ropars et al (2018).

e Clinical strains are less heterozygous than oak strains (Wilcoxon test, P=0.02).

f Wilcoxon test, P=0.01.

Using seqtk from SAMtools, we converted base calls in gVCF format to fasta format sequence and filtered bases that had quality scores below a phred-scaled quality score of 40 (equivalent to an error rate of 1 in 10,000). All genome sequences were mapped against the reference genome, and were therefore already aligned against it because insertions and deletions were excluded. fasta format alignment files were converted to phylip format using fa2phylip.pl (Bensasson 2018b).

### Verification of DNA sequence at multilocus sequence typing, mating type-like, and *ribosomal* DNA loci

We inferred the genotype calls at multilocus sequence typing (MLST), mating type-like (MTL), and ribosomal DNA (rDNA) loci for the type strain and the three oak strains using the genomic data generated above, and also verified these by independent DNA extraction, PCR, and sequencing. Purified genomic DNA was extracted as above, except that 100 units of lyticase was used to degrade the fungal cell wall for each preparation before DNA extraction. The DNA yield from each preparation was determined by fluorimetry using a Qubit 3.0 fluorometer (Thermo Fisher). The seven housekeeping MLST genes (*AAT1*α, *ACC1*, *ADP1*, *MPI1b*, *SYA1*, *VPS13*, and *ZWF1b*) used routinely for *C. albicans* strain typing were PCR-amplified and sequenced following the standard protocol (Bougnoux *et al.* 2003; Tavanti *et al.* 2003). The *C. albicans* MLST database (https://pubmlst.org/calbicans/) was used to determine allele identity (sequence type, ST). The mating type (a/α, a/a, or α/α) was determined by PCR using the method described by Tavanti *et al.* (2003). The complete internal transcribed spacer (ITS) region, encompassing ITS1, the 5.8S rRNA gene, and ITS2, was PCR-amplified directly from whole-yeast cell suspensions following the procedure and PCR parameters as described by James *et al.* (1996). The ITS region was amplified and sequenced using the conserved fungal primers ITS5 and ITS4 (White *et al.* 1990).

### Estimation of ploidy and analysis of heterozygosity

We developed a Perl script, vcf2allelePlot.pl (Bensasson 2018b), that uses R to visualize all the base calls that differ between a strain and the reference genome (SC5314_A22; Figure 1A and Figure S1). This vcf2allelePlot.pl was also used to identify loss of heterozygosity (LOH) regions as follows. The genome was divided into 100 kb nonoverlapping windows and LOH was called for windows where the proportion of heterozygous sites was <0.1%. We estimated the proportion of heterozygous sites after excluding low quality sites (phred-scaled consensus quality < 40), centromeres, annotated repeats, and sites in each window with over double the average genome-wide read depth for a strain. A large window size (100 kb) minimizes the chances of excluding a region of low heterozygosity due to sampling error. For example, for strains with 0.2% mean heterozygosity (Table 2), we expect around half the 10 kb windows to have <20 heterozygous sites per 10 kb region. Using a 100 kb window a random sample with 0.2% heterozygosity (expected 200 heterozygous sites), is less likely to dip below 0.1% (100 heterozygous sites). We used histograms generated in R (version 3.2.3 for this and all other statistical analysis) to decide the 0.1% threshold for the identification of LOH regions (Figure 1B and Figure S2).

For a comparison of heterozygosity among the clades defined by Ropars *et al.* (2018) for their clinical *C. albicans* strains, we used a standard analysis of variance with the “lm” function in R, and clade set as a factor with 18 levels (one level for each of the 17 defined clades and another level for strains of unknown clade). We tested whether oak strains (NCYC 4146, NCYC 4144, and NCYC 4145) showed higher heterozygosity than expected for their clade assignment (clades 4, 18, and unknown), by including “oak” as a separate clade level, and comparing this to a model where oaks were simply defined according to their clade. All models were checked using the model-checking plots of R as recommended in Crawley (2012). In addition, we used histograms to verify that heterozygosity levels were approximaetely normally distributed around the mean for all clades where at least 10 strains were available: clades 1, 2, 3, 4, 11, 13, and strains from unknown clades.

### Phylogenetic analysis and in silico chromosome painting

**Figure 2:**
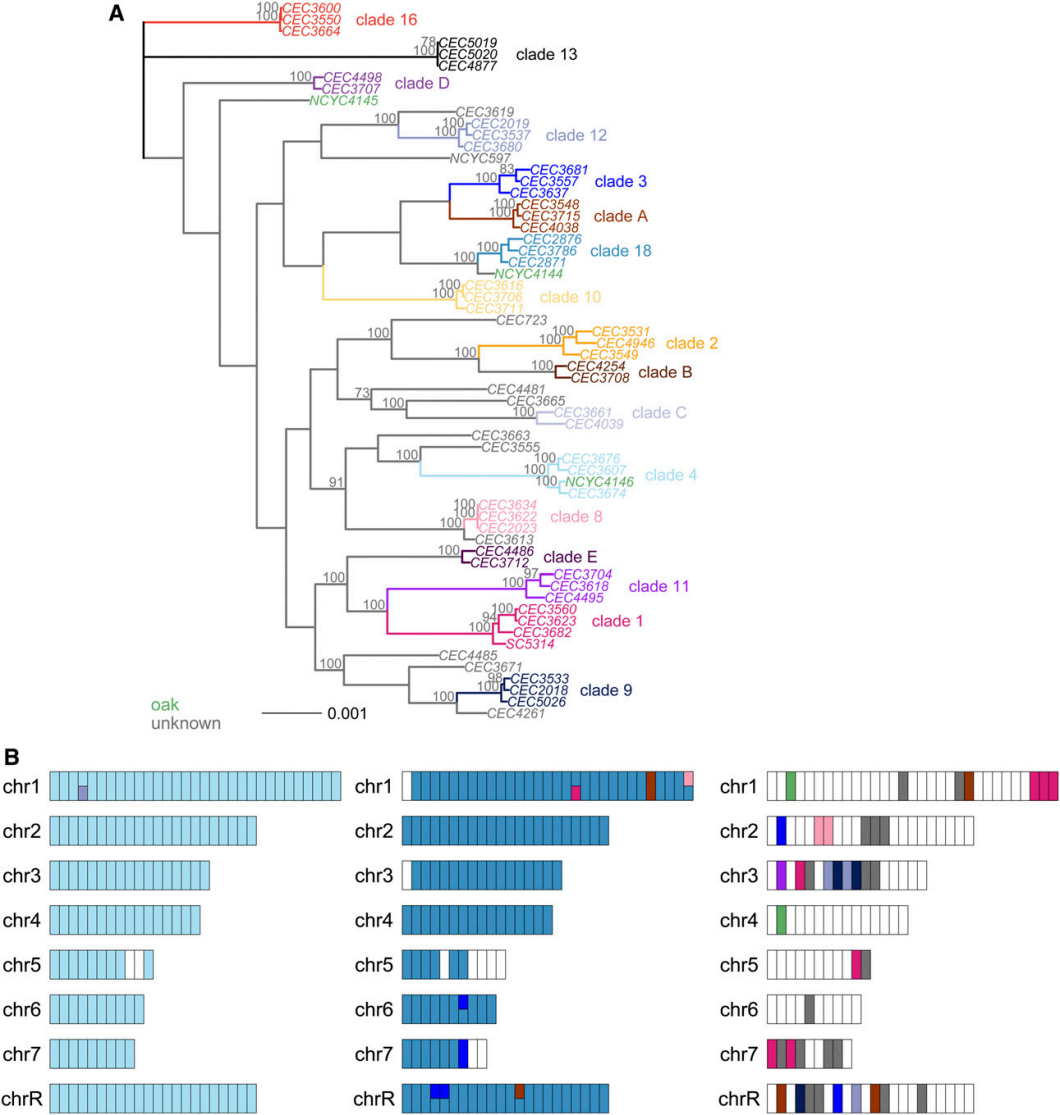
*C. albicans* from oak are more similar to clinical strains than to each other. Phylogenetic and pairwise sequence comparisons show that the oak strain NCYC 4146 is similar to clade 4 clinical strains, NCYC 4144 is similar to clade 18 clinical strains, and NCYC 4145 is diverged from the 17 clades sampled and 10 further strains of unknown clade. A. Genome-wide phylogeny. Maximum likelihood phylogenetic analysis of a whole-genome alignment (14,282,655 bp) shows that oak strains (green) are more closely related to clinical strains than they are too each other. Bootstrap support is estimated from 100 replicates and only values >70% are shown. The alignment contained 313,250 variable sites with at least one homozygous difference between strains. B. Painted chromosomes of oak strains. Most parts of the genomes of oak strains are more similar to clinical strains than to other oak strains (green). The genome of each oak strain was colored according to the clade assignment of the most similar strain for each 100 kb window in the genome using the colours for each clade shown in A. Regions are colored white if a strain sequence is diverged from all the other oak or clinical strains that we sampled (the proportion of sites differing is >0.028%). From left to right, NCYC 4146 in light blue is most similar to Clade 4 strains, NCYC 4144 in blue is most similar to Clade 18, and NCYC 4145 is mostly white and therefore diverged from all known clades.

We determined the relationships between strains using a maximum likelihood phylogenetic approach. For the phylogenetic analysis of the genome data from Ropars *et al.* (2018) shown in Figure 2A, we randomly selected three strains to represent clades where more than 3 strains were available, used all available strains for other clades and included the data for the three oak strains, the type strain (NCYC 597) and SC5314 (Muzzey *et al.* 2013). For all strains, genome sequence was generated by aligning each read to the SC5314 haplotype A reference while excluding all insertions (see above). Therefore all genome sequences were already aligned to this reference, and there was no need for further alignment. The resulting alignments for each chromosome are available from Bensasson (2018a). All eight chromosomes were concatenated into a single 14,282,655 bp genome-wide alignment using alcat.pl (Bensasson 2018b), which included 313,250 variable sites that included at least one homozygous difference between strains. We used the maximum likelihood phylogenetic approach implemented in RAxML (version 8.1.20; Stamatakis 2014) with a general time reversible evolutionary model, a γ-distribution to estimate heterogeneity in base substitution rates among sites (GTRGAMMA) and 100 bootstrap replicates. RAxML treats heterozygous base calls as missing data, so the resulting phylogeny is based on homozygous differences between strains.

To test whether a strain is similar to a single clade in all parts of its genome, we developed faChrompaint.pl (Bensasson 2018b) to “paint” chromosomes according to similarity to known clades. Several other tools already exist for painting chromosomes *in silico* to identify admixture between populations [reviewed in Schraiber and Akey (2015)]; however, these require phased haplotype data, which are not available for *C. albicans*. The faChrompaint.pl script takes fasta formatted whole-chromosome alignments as input, and divides the genome of a study strain into nonoverlapping windows (we set the window size to 100 kb). The script uses R to generate a plot with every window colored according to the clade assignment of the most similar strain in that window. The most similar DNA sequence was the one with the lowest proportion of differing sites. We defined clades using the clade assignments made by Ropars *et al.* (2018) for the strains confirmed in our phylogenetic analysis (Figure 2A), and colored windows green if their greatest similarity was to an oak strain sequence. If a strain shows high genetic divergence from all other strains, then similarity to a known clade does not necessarily imply recent common ancestry. We therefore filtered diverged regions by leaving those windows blank. More specifically, we did not color windows with over 0.028% divergence from known clades because most within-clade pairwise comparisons (90%) show divergence levels <0.028%, whereas most between-clade comparisons show divergence >0.028% (Figure S3).

### Data availability

DNA sequences determined for this study are available in the European Bioinformatics Institute, ENA as PRJEB27862, and whole-chromosome alignments that include sequences from Ropars *et al.* (2018) are available from https://github.com/bensassonlab/data (Bensasson 2018a; DOI: 10.5281/zenodo.1488207). Perl scripts are available at https://github.com/bensassonlab/scripts (Bensasson 2018b; DOI: 10.5281/zenodo.1488147). The type strain and *C. albicans* strains isolated from oak are available from the National Collection of Yeast Cultures (NCYC) in the United Kingdom. Supplemental material are available at Figshare: https://doi.org/10.25386/genetics.7367141.

## Results

### Phenotypically diverse strains of *C. albicans* from old oaks

We recently discovered *C. albicans* living on the bark of oak trees in a wood pasture in the New Forest (Table 1; Robinson *et al.* 2016). The three strains we isolated are available from the NCYC (NCYC 4144, NCYC 4145, and NCYC 4146). For several reasons, the data from Robinson *et al.* (2016) suggest that these three oak strains represent independent isolates and not laboratory contaminants or recent migrants from a single human or animal source. First, bark samples were collected into tubes using sterile technique from heights of at least 1.5 m above the ground, thus reducing the chances of direct contamination from animal manure. Second, we generated negative controls at the time of bark collection for two out of the three trees with *C. albicans* and they gave rise to no colonies after enrichment culturing. Subsequent DNA extraction and PCR amplification from these control plates were all also blank (Robinson *et al.* 2016). Third, the three trees harboring *C. albicans* were between 73 and 150 m apart, therefore any migration from animal manure or from humans to tree bark would have to occur in three separate events. Finally, trees with *C. albicans* had larger trunk girths and therefore were older than most of the 112 uncoppiced trees sampled across Europe [data from Robinson *et al.* (2016); Wilcoxon test, P=0.009] or in the New Forest (Table 1; Wilcoxon test, P=0.04). There is no reason to expect a greater level of migration from animals on old trees unless *C. albicans* are able to live on oak for many years.

*C. albicans* that were isolated from plants in the past were pathogenic in mammals, but did not differ from each other phenotypically (van Uden *et al.* 1956; Di Menna 1958). To test whether the *C. albicans* strains from oak also resembled each other phenotypically, we tested the growth of oak strains under the standard conditions described in Kurtzman *et al.* (2011). All three oak strains were able to grow at elevated temperatures (37–42°; Table S1), suggesting that they would be able to survive temperatures in a mammalian host. The oak strains also produced well-formed, irregularly branched pseudohyphae when grown on either corn meal agar or potato dextrose agar, and grew in 60% glucose, showing that they were highly osmotolerant. The phenotypes of the three oak strains differed from each other in that NCYC 4145 and NCYC 4146 displayed salt tolerance by growing in the presence of 10% NaCl, but NCYC 4144 did not (Table S1). The results of further growth tests on agar, in broth, and under other conditions were as previously described for *C. albicans* (Lachance *et al.* 2011), with the exception that one oak strain (NCYC 4144) formed pseudohyphae in YM broth and did not grow on soluble starch (Table S1).

In addition, the three *C. albicans* strains from oak must be able to survive the cool temperatures and other characteristics of the oak environment because they lived there until they were isolated from bark. They also survived enrichment, storage, and culturing conditions, which include growth at room temperature (30°) in a liquid medium containing chloramphenicol (1 mg/liter) and 7.6% ethanol, on selective plates with a sole carbon source of methyl-α-D-glucopyranoside (Sniegowski *et al.* 2002), storage at 4°, and as 15% glycerol stocks at −80° (Robinson *et al.* 2016).

### Oak *C. albicans* are mostly diploid with some LOH

Clinical strains of *C. albicans* are predominantly diploid (Hickman *et al.* 2013; Hirakawa *et al.* 2015), yet aneuploidy is often observed and may be an important mechanism for adaptation (Li *et al.* 2015; Todd *et al.* 2017). In addition, clinical strains undergo LOH with even greater frequency than changes in aneuploidy (Ford *et al.* 2015; Ropars *et al.* 2018), and LOH can affect mating type and other phenotypes (Bougnoux *et al.* 2008; Forche *et al.* 2011; Ford *et al.* 2015; Hirakawa *et al.* 2015). We therefore tested for aneuploidy and LOH in oak strains using a standard base calling approach for estimating ploidy and LOH from genome sequence, and validated our methods by using them to identify known aneuploidy and LOH events in clinical and laboratory strains (Supplemental Results).

The base calling approach we used (the B allele approach; Teo *et al.* 2012; Yoshida *et al.* 2013; Zhu *et al.* 2016) estimates the minimum ploidy of a yeast strain by examining the base calls of short read genome data mapped to a reference genome (SC5314 haplotype A). In a haploid genome or a homozygous diploid, base calls at the single nucleotide polymorphic (SNP) sites relative to the reference genome will all differ from the reference genome, so the proportion of base calls that differ from the reference will be approximately equal to 1 (*e.g.*, chromosome 5 of the oak strain NCYC 4144 in Figure 1A). In a diploid, the proportion of base calls differing from the reference will be approximately equal to 1 at homozygous SNP sites or 0.5 at heterozygous SNP sites (*e.g.*, the oak strain NCYC 4146 in Figure 1A). In triploids, the proportion of calls differing from the reference will be 0.66 and 0.33 at heterozygous sites (*e.g.*, chromosome R of NCYC 4144 in Figure 1A) and so on. It is also possible to detect aneuploidy by comparing read depth between chromosomes. However, we use a base calling approach because read depth approaches are sensitive to biases when genomes are fragmented enzymatically (Marine *et al.* 2011; Quail *et al.* 2012; Teo *et al.* 2012), as they were in this study (see *Materials and Methods*).

For all three oak strains, the proportion of base calls differing from the reference is 0.5 at most heterozygous sites and therefore they are predominantly diploid (Figure 1A). NCYC 4146 appears to be euploid, but NCYC 4144 and NCYC 4145 show evidence of trisomy on chromosome R. The type strain and some other clinical strains are also trisomic for chromosome R or have three copies of its right arm (Hirakawa *et al.* 2015; Ropars *et al.* 2018; Figure S1). The right arm of chromosome R may therefore exist in three copies with appreciable frequency in natural strains of *C. albicans*. Trisomy of chromosome R can result in slow growth in the laboratory (Hickman *et al.* 2015), and has been associated with resistance to triazoles (Li *et al.* 2015).

The oak strains also showed several chromosomal segments (light blue in Figure 1A) that were homozygous (heterozygosity < 0.1% in Figure 1B). Read depth in regions with low heterozygosity is similar to that in the rest of the genome (Figure S4), therefore these regions do not represent deletions or the loss of a chromosome. They are regions that have undergone recent LOH. Consistent with the proposal that aneuploidy persists for less time in *C. albicans* populations than LOH regions (Ford *et al.* 2015), we observe a greater number of LOH regions than aneuploidy events for every oak and clinical strain (Figure 1 and Figure S1).

One oak strain (NCYC4144) is homozygous across the whole of chromosome 5 on which the mating locus is situated. Analysis of whole-genome data, and confirmation by independent PCR and sequencing shows that this strain is homozygous for the *a* allele at the *C. albicans* mating locus (a/a) and therefore could potentially mate with strains that are homozygous for the opposite mating type (α/α). Whole-chromosome homozygosis is not an unusual mechanism by which natural strains of *C. albicans* become homozygous at the MTL locus (Hirakawa *et al.* 2015).

### Oak strains are phylogenetically diverse

Most clinical strains of *C. albicans* belong to a small number of genetically diverged clades, have a global distribution and live alongside each other in the same human populations (Bougnoux *et al.* 2006; Odds *et al.* 2007). Phylogenetic comparisons between oak and clinical strains can be used to determine whether oak strains form distinct populations that differ genetically from clinical strains, as they do in *S. cerevisiae* (Almeida *et al.* 2015; Peter *et al.* 2018). Using whole-genome phylogenetic analysis, we therefore compared oak strains to clinical strains from 17 of the most abundant *C. albicans* clades (two or three strains from each clade) and 10 further strains that are from unknown clades [data from Ropars *et al.* (2018); Figure 2A].

All three oak strains are phylogenetically distinct from each other and more similar to clinical strains than they are to each other (Figure 2). One strain from oak (NCYC 4146) belongs to clade 4, which is one of the most abundant clades, with a known worldwide distribution (Figure 2). This clade assignment is also supported by the region of LOH that we see on the right arm of chromosome 3 (Figure 1A), which Ropars *et al.* (2018) showed was common to all clade 4 strains. Another oak strain (NCYC 4144) is similar to clade 18 strains (Figure 2), which have been isolated from east Asia and Portugal (Shin *et al.* 2011; Ropars *et al.* 2018). In contrast, the oak strain NCYC 4145 is unlike any of the 27 clinical lineages represented in our phylogenetic analysis (Figure 2). Genome data are only available for two strains of *C. albicans* from animals, and both were from starlings in France belonging to clade 16; none of the oak strains are similar to these (CEC3550 and CEC6000 in Figure 2A). Phylogenetic analysis of MLST loci also shows that oak strains are no more similar to 28 strains from wild and domesticated animals from the United States than they are to strains from humans (Figure S5).

By considering only whole-genome phylogenies, or data pooled across many loci, we could miss genetic similarities between the oak strain of unknown clade (NCYC 4145) and known clades. For example, if NCYC 4145 is a parasexual hybrid between two strains of known clade, its phylogenetic placement could suggest it is distinct from both parents. To detect regional genomic similarities, we therefore compared each oak strain genome to every other oak and clinical strain genome by painting their chromosomes *in silico* according to the clade assignment of similar strains (Figure 2B). This analysis of NCYC4145 in short (100 kb) blocks across the whole genome suggests that it is genetically diverged from all sampled clades (Figure 2B). Compared with other oak strains (Figure 2B) or with clinical strains from known clades (Figure S6, a–q), NCYC4145 had more regions that were diverged from other sampled strains. In this, NCYC4145 is similar to clinical strains that are from clades only represented by a single strain (Figure S6r). Fine-scale chromosome painting analysis shows that the clade assignment of the other two oak strains are consistent across almost all parts of the genome (Figure 2B). As expected for a predominantly asexual species, we also see this consistency in clade assignment for all clinical strains of known clade (Figure S6, a–q).

### High heterozygosity on oaks

Levels of heterozygosity are high in *C. albicans* compared with the levels expected from a sexually reproducing species (Bougnoux *et al.* 2008), and this is likely to be the ancestral state for the species (Ropars *et al.* 2018). In the absence of mating and mitotic recombination, the haplotypes within a lineage will diverge as they accumulate mutations, whereas in sexual species, recombination can lead to increased similarity between alleles (Birky 1996; Halkett *et al.* 2005). Because heterozygosity correlates with fitness (Hickman *et al.* 2013; Hirakawa *et al.* 2015; Ciudad *et al.* 2016), virulence, and niche breadth (Ropars *et al.* 2018), we compared levels of heterozygosity between the three oak strain genomes and 180 of the clinical strain genomes analyzed by Ropars *et al.* (2018). High-quality sequence was available for ∼14 million sites in the genome for all oak and clinical strains, and we estimated the proportion of these sites that were heterozygous (Table 2 and Table S3). We excluded two clinical strains of *C. albicans* (CEC3678 and CEC3638 from clades 1 and 3) from the data set of Ropars *et al.* (2018) because these had fewer than 9 million high-quality sites.

Levels of heterozygosity are higher in the oak strains (0.61, 0.62, and 0.77%) than they are for 180 clinical strains (mean, 0.48%; Table 2; Wilcoxon test, P=0.02). In contrast, the clinical strain of *C. albicans* (NCYC 597) that we studied in the same sequencing batch as the oak strains showed a level of heterozygosity (0.48%) that was similar to other clinical strains (Table 2), suggesting that high heterozygosity is not an artifact of the sequencing methods used in this study. Furthermore, we excluded all sites with low quality sequence (with an expected error rate over 1 in 10,000), and the oak strains did not differ from clinical strains in the amount of high-quality sequence analyzed (Wilcoxon test, P=0.2; Table S3).

Levels of heterozygosity can vary within genomes because of recent LOH, and higher heterozygosity at centromeres that evolve faster than other genomic regions in *C. albicans* (Padmanabhan *et al.* 2008; Ropars *et al.* 2018). The number of heterozygous sites could also be overestimated in repetitive regions as a result of the mismapping of short reads to the reference genome. We therefore estimated levels of heterozygosity after filtering out LOH regions, centromeres, known repeats (using the reference genome annotation), and sites with over double the mean genome-wide read depth that could represent unannotated repeats. Even after applying this filter, levels of heterozygosity are higher for oak strains (mean, 0.70%) than they are for clinical strains (mean, 0.56%; Wilcoxon test, P=0.01; Table 2). This difference in heterozygosity results from heterozygosity at thousands of sites across the genome (Table S3). For example, the strain NCYC 4145 is heterozygous at 0.78% of the 11.4 million sites we studied after filtering, and therefore has 20,000 more heterozygous sites than expected for a clinical strain, and 5000 more heterozygous sites than expected for the most heterozygous of the 180 clinical strains (0.73% for CEC5026 from clade 9; Table S3).

There is recent evidence that *C. albicans* clades can differ in levels of heterozygosity (Ropars *et al.* 2018). For example, 35 of the 180 clinical strains are clade 13 strains, which are unusually homozygous compared with other clades (Ropars *et al.* 2018). We also observe substantial differences among the clades of clinical strains in levels of heterozygosity even after filtering LOH regions, centromeres, and repeats (ANOVA: $F_{17,162}=240,$ $P<2\times 10^{-16}$). Clade 13 strains are unusually homozygous (mean, 0.33%), compared to the average for clinical strains (mean, 0.56%), while clades 9, 10, and 16 are the most heterozygous. Could oak strains be highly heterozygous simply because they belong to highly heterozygous clades? ANOVA shows that oak strains are more heterozygous than clinical strains even when comparing them to strains with similar clade assignments (Table 2; ANOVA: $F_{1,165}=22,$ $P=6\times 10^{-6}$), and when excluding the homozygous clade 13 strains from the comparison (ANOVA: $F_{1,131}=18,$ $P=5\times 10^{-5}$). The oak strains NCYC 4146 and NCYC 4144 are more heterozygous than any other strain from their respective clades 4 and 18, while NCYC 4145, which we could not assign to a clade, was more heterozygous than any of the 180 clinical strains (Table 2).

The three oak strains might also differ from the sample of 180 clinical strains at their MTL locus (Table 2). The clinical strains show a ratio of 176 a/α:3 α/α:1 a/a genotypes at their MTL locus, therefore it is surprising to encounter an a/a genotype in a sample of only three oak strains (Fisher’s exact test, P=0.03). To exclude the possibility that this difference could explain the higher levels of heterozygosity of oak strains, we also compared the oak strains to a sample of 22 clinical strains with an MTL genotype ratio that is more similar to that seen in oak, 10 a/α:6 α/α:6 a/a (Table S2; data from Hirakawa *et al.* 2015). Levels of heterozygosity are also higher for oak strains (0.61–0.77%) than for any of these 22 clinical strains (0.35–0.60%; Table S2; Wilcoxon test, P=0.0009). Once more, after filtering out LOH regions, centromeres, and repeats, oak strains are more heterozygous (mean, 0.70%) than clinical strains (mean, 0.60%; Table S2; Wilcoxon test, P=0.003). Finally, to address the possibility that oak strains have undergone less LOH in fast-evolving regions than clinical strains, and that this could therefore account for their higher heterozygosity, we also measured heterozygosity at 948,860 nucleotide sites that were high-quality, non-LOH, nonrepetitive, and noncentromeric in all 22 oak and clinical strains from Table S2. At these sites, oak strains were more heterozygous (mean, 0.72%) than clinical strains (mean, 0.61%; Wilcoxon test, P=0.01; Table S2). In all analyses across multiple data sets, oak strains therefore show a small but statistically significant elevation in their levels of heterozygosity.

## Discussion

### High diversity of *C. albicans* from oak trees

Phylogenetic analyses and fine-scale genome-wide DNA sequence comparisons show that all three strains from oaks from a single woodland site belong to distinct clades that show substantial genetic divergence (Figure 2). Two of the oak strains (NCYC 4146 and NCYC 4144) belong to clades 4 and 18, and both of these clades have been encountered in European clinical strains as well as from other continents (Odds *et al.* 2007; Shin *et al.* 2011), while one oak strain (NCYC 4145) is from a rare clade that has not yet been described. *C. albicans* strains from wild and domestic animals from Germany, northwestern Europe, and the United States show greater similarity to strains from humans than to each other, suggesting migration of *C. albicans* strains between humans and other animals (Edelmann *et al.* 2005; Jacobsen *et al.* 2008; Wrobel *et al.* 2008). Phylogenetic analysis at MLST loci also shows that the oak strains are no more similar to animal strains than they are to strains from humans [Figure S5; data from Wrobel *et al.* (2008)]. Our findings therefore suggest that migration between humans and woodland environments is also possible.

Could the oak strains represent a random set of recent migrants from humans to the oak environment? This possibility is difficult to reconcile with the different levels of heterozygosity seen in oak strains compared to 180 clinical strains (Table 2). The higher heterozygosity of oak strains remains when we account for the differences between clades (Table 2; ANOVA: $F_{1,165}=22,$ $P<6\times 10^{-6}$), and when we compare to a different set of 22 clinical strains with more MTL homozygous strains (mean, 0.60%; Table S2; Wilcoxon test, P=0.003). In other *Candida* species, high genome-wide heterozygosity has arisen as a result of hybridization between subspecies (Pryszcz *et al.* 2014). Chromosome-painting analysis shows that interclade hybridization is not the source of the high heterozygosity seen in *C. albicans* from oak (Figure 2B).

High levels of heterozygosity probably represent the ancestral state for *C. albicans*, because most closely related *Candida* species also show high levels of heterozygosity (Ropars *et al.* 2018), which is expected for asexual species (Birky 1996). Consistent with the idea that low heterozygosity represents the derived state for the species, strains from the most homozygous clade (clade 13) have sustained many loss-of-function mutations to their genes, grow poorly under a range of laboratory conditions, and have a restricted niche compared with other clades (Ropars *et al.* 2018). In *C. albicans*, there is also evidence that high heterozygosity is maintained through natural selection of heterozygotes that do not expose recessive deleterious mutations. Natural strains with high genome-wide heterozygosity show higher laboratory fitness (Hirakawa *et al.* 2015), while homozygous diploid strains grow poorly compared to heterozygous strains (Hickman *et al.* 2013) and LOH across even small genomic regions can lead to negative fitness consequences under stress (Ciudad *et al.* 2016). Therefore, the high genome-wide heterozygosity of *C. albicans* from oaks suggests that this is not necessarily a transient niche, and that these strains have not been subject to less natural selection than clinical strains.

The phenotypes of these oak strains also suggest that they have not narrowed their niche relative to clinical strains. Unlike the relatively homozygous clade 13 strains, which do not grow well at high temperature (42°) and have only been isolated from the genital niche in humans (Ropars *et al.* 2018), the oak strains grew well at 42°, suggesting that they would not be restricted to a superficial niche in mammals. Past studies of *C. albicans* from grass and shrubs showed that environmental isolates were able to grow in rabbits and kill them within days (van Uden *et al.* 1956; Di Menna 1958). We do, however, observe more phenotypic differences among oak strains (Table S1) than in these early studies (van Uden *et al.* 1956; Di Menna 1958). This functional diversity could reflect the differences in mating type, heterozygosity, or clade among oak strains.

### *C. albicans* lives on old oaks in an ancient wood pasture

*C. albicans* from oak differ from clinical strains in that they are unusually heterozygous, showing heterozygosity at thousands of sites more than expected for clinical strains (Table 2 and Table S3). Furthermore, the three oak strains were genetically diverged from each other (Figure 2), which implies that they do not represent laboratory contaminants from a human. Humans only rarely carry more than one distinct strain of *C. albicans*, and strains from multiple clades are especially rare (Bougnoux *et al.* 2006). In addition, these strains were isolated from three different trees that were more than 75 m apart, from bark over 1.5 m above the ground, and alongside negative controls that were clear (Robinson *et al.* 2016). The genetic divergence between oak strains also implies that these oak trees were not colonized as a result of migration from a single animal in the woods. It is rare for domestic or wild animals to carry multiple strains of *C. albicans* and it is especially rare for these to belong to different clades (Wrobel *et al.* 2008).

Experiments with *S. cerevisiae* and *Saccharomyces paradoxus* show that yeast can grow on the nutrients in oak bark (Kowallik *et al.* 2015) and live in forest soils for months or years (Anderson *et al.* 2018). *C. tropicalis* are also reliably isolated from trees in Canada over a period of several years (Carvalho *et al.* 2014). Intriguingly, *C. albicans* only occurred on some of the oldest oak trees compared with our European sample (Wilcoxon test, P=0.009; Robinson *et al.* 2016) or with other trees in the New Forest (Table 1). If oak *C. albicans* are merely recent migrants from animals, then they should occur on young and old trees with equal probability. In contrast, we expect a greater prevalence of *C. albicans* on old trees if they resemble other yeast species that colonize oak trees gradually and persist there over a period of decades (Robinson *et al.* 2016). Therefore, the occurrence of phylogenetically diverse *C. albicans* on unusually old oak trees suggests that it could live in this environment for decades, and that it is not an obligate commensal of warm-blooded animals.

If *C. albicans* can stably inhabit a woodland environment, then why have they only been isolated from trees on a handful occasions (Lachance *et al.* 2011; Robinson *et al.* 2016)? For example, three surveys of trees did not discover *C. albicans* but reported the isolation of other *Candida* species, and therefore could have detected *C. albicans* if it were present (Maganti *et al.* 2011; Charron *et al.* 2014; Sylvester *et al.* 2015). More recently, a larger survey did yield several *C. albicans* strains on fruit, soil, and plant matter in northern North America (Opulente *et al*. 2018), so the lack of *C. albicans* environmental isolates could be due to a lack of past sampling effort. In addition, if past surveys for woodland yeast did not target old trees, it is possible that *C. albicans* could have been missed.

The comparison between other human pathogenic fungi, such as *Cryptococcus*, and their wild relatives on trees is important for understanding disease emergence (Gerstein and Nielsen 2017). Furthermore, study of the natural enemies of *C. gattii* that occur where it resides on plants could lead to the development of new antifungal drugs (Mayer and Kronstad 2017). If *C. albicans* is not an obligate commensal of warm-blooded animals, then comparisons between clinical and free-living strains of *C. albicans* will also be important for understanding its commensalism and pathogenicity. A major limitation in this endeavor is that very few *C. albicans* strains are available for study from nonanimal sources. The few isolates that have been obtained were from a broad range of sources (van Uden *et al.* 1956; Di Menna 1958; Lachance *et al.* 2011; Robinson *et al.* 2016), and general environmental sampling for fungal pathogens can be challenging (Gerstein and Nielsen 2017). In the future, the targeting of old trees could lead to improved environmental sampling success.
